# Theory of Gout

**Published:** 1814-01

**Authors:** 


*To the Editor of the Medical and Physical Journal.*

SIR,

I KNOW not whether the following theory of gout be original or not: much of what we read we often identify with our own views of particular subjects; and when engaged in contemplating them, we very often apply the reasoning and facts of others as if they were our own, for the explaining and developing of any difficulties which may occur for resolution.

In this way it is very possible, and quite as probable, that I may not be a discoverer; but I shall be well enough pleased if, under the satisfactory illusion of being one, from inferences to be deduced, the following theory shall be acknowledged to be warranted, and that, by an explanation of the causes of a particular disease, which it is said has hitherto baffled explanation, such disease shall often be avoided; and, what is almost of as much importance, those who are afflicted with it shall cease to hope for cure by means ill calculated to effect it. And if the following theory shall be deemed well or rationally founded, such persons will also be taught, what indeed is well known, although the reason for its efficacy in preventing, if not in curing the disease, may not be so generally understood, that temperance, and temperance only, is the only fundamental preventive, and the only rational cure of this disease where at all curable.

Gout then I conceive to be inflammation arising from that lesion of the terminating vessels, by which they are prevented duly fulfilling their assigned offices of secretion and apposition.

I need hardly pursue this notion. Many of your ingenious correspondents will easily measure the effects of increased action of the arterial and secreting system upon the exquisitely fine vessels engaged in that wonderful part of our frame.

Impulse upon a set of vessels in a greater degree than they are calculated to bear, must have the effect of deteriorating them in their weakest part: that part is where their organisation is the most delicate, and therefore the most fragile. Let the heart be incited, whether by intense or rapturous thought; let the whole system be perpetually immersed in floods of delight beyond the common and accustomed bliss of nature, gout may supervene; but as all this is within the possibilities *naturally* incidental to our organisation, it does not give way: secretion and apposition may yet go on. Let, however, alcohol be superadded to all these, cardiac, and consequently arterial action is increased; the system, throughout all its indescribably fine ramifications, partakes of the accelerated action; and, borne down by calls upon, and solicitations of, its powers, it incontestibly yields to the unnatural impulse it experiences, and, being the finest, those vessels which are destined to ultimate secretion and apposition are the first to break up. The excited action goes on, but secretion and apposition are weakened, disappointed, or destroyed. That set of vessels which, in our nicely adjusted machinery, was destined to perform certain vital functions, can no longer act; the parts depending upon that action for their vitality become gorged; arterial action may have tended to the formation of the ultimate matter, but the organisation adapted for apposition is weakened or destroyed: even secretion or formation of the necessary matter is at length interrupted or prevented. The whole of these fine functions become disturbed, and hence the premature formation formation and apposition of crude bone, where, in a state of heat, there should have been colorless blood, or membranous apposition. Where inflammation had already occurred from gorgement with matter which absorbents had been unable to take up, it is occasioned by the deposition of matter alien both in place and substance to its true and natural designation and character. Hence chalk stones; and hence probably that ultimate destruction of vital action known by the name of gout in the stomach, an hypothetical disease never detected in the stomach as in membranes about the feet and hands.

Had I leisure, I would willingly have resolved what I have said into the form of aphorisms; but they would repeat so many truisms, while the conclusion is itself simple and to me self-evident, that I have been induced to prefer thus rudely recording my view of the disease of gout in your valuable Journal. I am with great respect,

